# TSPO: An Evolutionarily Conserved Protein with Elusive Functions

**DOI:** 10.3390/ijms19061694

**Published:** 2018-06-07

**Authors:** Frederick Bonsack, Sangeetha Sukumari-Ramesh

**Affiliations:** Department of Neurosurgery, Medical College of Georgia, Augusta University, 1120 15th Street, Augusta, GA 30912, USA; fbonsack@augusta.edu

**Keywords:** TSPO, 18 kDa translocator protein, steroidogenesis, glial activation, neuroimaging

## Abstract

TSPO (18 kDa translocator protein) was identified decades ago in a search for peripheral tissue binding sites for benzodiazepines, and was formerly called the peripheral benzodiazepine receptor. TSPO is a conserved protein throughout evolution and it is implicated in the regulation of many cellular processes, including inflammatory responses, oxidative stress, and mitochondrial homeostasis. TSPO, apart from its broad expression in peripheral tissues, is highly expressed in neuroinflammatory cells, such as activated microglia. In addition, emerging studies employing the ligands of TSPO suggest that TSPO plays an important role in neuropathological settings as a biomarker and therapeutic target. However, the precise molecular function of this protein in normal physiology and neuropathology remains enigmatic. This review provides an overview of recent advances in our understanding of this multifaceted molecule and identifies the knowledge gap in the field for future functional studies.

## 1. Introduction

Translocator protein (18 kDa, TSPO), which was initially designated as the peripheral-type benzodiazepine receptor (PBR), was identified in 1977 when investigators were searching for peripheral tissue binding sites for benzodiazepines, one of the most widely available class of drugs prescribed to treat patients with anxiety, convulsions, or insomnia [1]. TSPO was discovered in the kidney as a diazepam-binding site [1], and the initial characterization of these benzodiazepine-binding sites outside the brain led to their assignment as ‘peripheral-type’ benzodiazepine receptors (PBR), to distinguish them from the central benzodiazepine receptors (CBR). Two types of benzodiazepine binding receptors have been identified in mammalian tissues, the central-type receptors (CBR) and peripheral-type benzodiazepine receptors (PBR). CBR are located on neurons and found coupled to GABA receptors, regulating the GABA-regulated chloride channels, [2,3] while PBR have a much more ubiquitous distribution [4,5]. PBR is primarily located on the outer membrane of mitochondria and found to be abundant in steroid-synthesizing cells [4]. It is also reported that PBR is pharmacologically and structurally distinct from the CBR [6,7]. Although the name ‘PBR’ was widely accepted previously, multiple other names have also been used to refer this protein such as mitochondrial benzodiazepine receptor, mitochondrial diazepam-binding inhibitor (DBI) receptor complex and PK11195-binding sites [8,9]. However, regardless of its interactions with other proteins or ligands, PBR was renamed as 18 kDa Translocator Protein (TSPO) in 2006 by the HUGO Gene Nomenclature Committee [4] reflecting its putative function in protein or ligand transport/translocation.

## 2. Evolutionary Conservation of TSPO

The primary coding sequence of TSPO is highly conserved throughout evolution, from bacteria to humans [8,10,11,12], and predicts a tryptophan-rich hydrophobic protein with five transmembrane domains. The functional TSPO protein occurs throughout the phylogenetic spectrum and the cDNA for TSPO has been cloned from various species including humans [10,13]. Notably, the rat TSPO replaced the activity of its bacterial homolog in *Rhodobacter sphaeroides* [14], indicating evolutionarily conserved functions of TSPO, although these proteins share only about 30% amino acid identity. In the human genome, the *TSPO* gene is localized to the chromosome 22, within the band 22q13.31 as a single copy and the mRNAs of human and mouse TSPO translate to closely related proteins having 169-amino acid residues with 81% sequence similarity [15,16,17]. TSPO is widely expressed throughout the body and the binding sites for TSPO ligands have been identified in tissues such as heart, kidney, and liver [7]. Further, TSPO is found enriched in tissues in which steroids are synthesized such as adipose tissue and adrenal cortex. In the CNS, the basal expression of TSPO is low and is restricted mostly to glial cells [18,19]. TSPO is a nuclear-encoded protein and at the subcellular level, TSPO is mainly localized in the outer mitochondrial membrane [20], reflecting a key role of TSPO in cellular functions related to mitochondria. Of note, TSPO has been implicated in a wide range of cellular processes including, but not limited to, proliferation and differentiation, apoptosis, immunomodulation, oxidative stress, and mitochondrial physiology [20,21,22,23,24]. A recent study employing microarray analysis of gene expression in a glioblastoma cell line, U118MG, upon treatment with TSPO ligand, PK11195 demonstrated that the mitochondrial expression of TSPO could be a part of mitochondria-to-nucleus signaling pathway resulting in modulation of nuclear gene/transcription factor expression and altered cellular functions [24]. Furthermore, apart from the expression of TSPO in the mitochondrial membrane, it has also been localized in the plasma membrane as well as in nuclear/peri-nuclear areas [25,26,27]. However, the precise molecular function of TSPO as well as its mode of action, whether it operates as molecular receptor or sensor remains largely unclear. In addition, the role of TSPO specific to a particular subcellular localization in normal physiology and pathology needs investigation.

## 3. TSPO and Steroidogenesis

Consistent with the abundance of TSPO expression in steroid producing tissues, TSPO was considered to be essential for the translocation of cholesterol from the outer mitochondrial membrane to the inner mitochondrial membrane, which is regarded as a rate-limiting step for steroidogenesis [21,28,29,30]. It was proposed that TSPO transports cholesterol across the outer mitochondrial membrane to a steroidogenic enzyme, CYP11A1 [31], which converts cholesterol into pregnenolone, a common precursor for steroids. Of note, synthetic ligands of TSPO such as PK11195 and Ro5-4864 stimulated steroidogenesis and neuro steroidogenesis both in vitro and in vivo [21,28,29,32,33,34]. Further, recent studies demonstrated a positive correlation between the TSPO ligand residence time (the period for which the ligand interacts with its target, TSPO) and its neurosteroidogenic efficacy [35,36]. In addition, the benzodiazepine, Ro5-4864 and the isoquinoline carboxamide, PK11195 exhibit nanomolar affinity for the TSPO and have distinct binding sites on TSPO [37,38]. The thermodynamic studies indicated that the [^3^H]-PK11195 binding to TSPO is entropy driven, in contrast, the [^3^H]-RO5-4864 binding is enthalpy driven [39]. Therefore, PK-11195 is being considered as an antagonist of TSPO, and RO5-4864, an agonist or a partial agonist [39] and both have been utilized extensively as prototypical pharmacological tools for characterizing TSPO and its molecular function. Apart from a putative role of TSPO in steroidogenesis, earlier studies have also reported embryonic lethality of TSPO knockout mice [40] implicating a key role of TSPO in normal physiology and development. In contrast, recent studies demonstrated that genetic deletion of TSPO in different cell types had no effect on cellular viability [41]. More importantly, the global TSPO knockout mice that were developed by two independent research groups by Cre-lox technology were viable and exhibited unaltered steroidogenesis [42,43]. Additionally, studies employing a transgenic mouse with conditional TSPO deletion in Leydig cells demonstrated that TSPO was not essential for testosterone production [44]. However, very recent studies demonstrated that global TSPO deletion alter adrenocorticotropic hormone-induced plasma corticosteroid concentrations [45] and TSPO deletion-mediated effects exacerbate with aging [46]. Altogether, the emerging evidence suggests elusive and conflicting roles of TSPO in mammalian cells that warrant further investigation. TSPO ligands augmented steroid hormone production in several different steroidogenic cell types [22,32,47]. Given the cytoprotective effects of steroids, the TSPO ligands have been proposed as therapeutic agents to augment steroid levels in the brain as well as in the reproductive system and the pharmacological agents have been extensively used to elucidate the physiological relevance of TSPO. However, genetic studies showed that the pharmacological effect of TSPO ligand, PK11195 on the induction of steroidogenesis is not mediated through TSPO in MA-10 mouse Leydig tumor cells [48] implicating the possible off-target effects of synthetic ligands and thereby emphasizing the essentiality of genetic cellular and animal model systems in elucidating the TSPO ligand-mediated effects on cellular functions.

## 4. TSPO and Mitochondrial Functions

Notably, recent studies employing genetic approach demonstrated a significant shift in mitochondrial homeostasis in *Tspo*^−/−^ fibroblasts that could affect multiple mitochondrial functions [49]. Of note, the ligands of TSPO regulate the mitochondrial permeability transition pore (MPTP) functioning and an association of TSPO with MPTP had been suggested previously [50]. However, recent studies demonstrate that TSPO plays no role in the regulation of MPTP. Further, both endogenous, as well as synthetic ligands of TSPO, do not regulate MPTP activity through TSPO implicating that TSPO-mediated modulation of mitochondrial functions could be independent of the regulation of MPTP [51]. Recent studies have also reported that genetic deletion of TSPO results in an increase in mitochondrial fatty acid oxidation in steroidogenic cells [52] and a decrease in oxygen consumption rate (OCR) in microglia [43] and fibroblasts [49] but the underlying molecular mechanisms of these findings and their relevance to the overall mitochondrial function require further investigation.

## 5. TSPO and Endogenous Ligands

Various endogenous TSPO ligands have been proposed such as cholesterol, Diazepam Binding Inhibitor (DBI), and porphyrin and the endogenous ligands bind TSPO with different affinities. Cholesterol is a potent ligand of TSPO with nanomolar affinity [53] and it binds to the cholesterol recognition amino acid consensus sequence in the carboxyl-terminal end of TSPO. In contrast, DBI has micromolar affinities for both TSPO and CBR and was initially described based on its ability to interact with CBR and regulate GABAergic transmission [54]. DBI and its proteolytic products can stimulate steroidogenesis by interacting with TSPO [55] and are widely distributed in the CNS and peripheral steroidogenic cells [56,57,58,59,60].

TSPO from all species studied bind porphyrins or cyclic tetrapyrroles (protoporphyrin IX (PPIX), mesoporphyrin IX, deuteroporphyrin IX, heme, and hemin) [61,62,63,64] and porphyrins exhibit high (nM) affinity for TSPO but not for CBR [62,65]. The concept that porphyrins are endogenous ligands is consistent with the mitochondrial location of TSPO since the mitochondria play a key role in porphyrin metabolism [66]. Many porphyrins are naturally occurring and one of the best-known porphyrins is heme, the red blood cell pigment and a cofactor of oxygen- binding protein, hemoglobin. In both eukaryotes and prokaryotes, TSPO interacts with heme and its immediate precursor PPIX [67]. Though it has been suggested that TSPO is a porphyrin transporter, no published experimental evidence supports this claim. Instead of acting as a transporter it is proposed that TSPO binds PPIX as a regulatory cellular protection mechanism against oxidative stress otherwise generated by the free form of these porphyrins. Detergent-purified TSPO can bind porphyrins in vitro, including PPIX and hemin (oxidized heme) [68,69]. Further, Rat TSPO can bind PPIX in vivo and the binding was demonstrated with positron emission tomography [70]. However, the mechanisms involved in the porphyrin/heme binding to the TSPO remain to be determined.

It was proposed that TSPO could sequester free heme in cells and catabolize/degrade excess PPIX in conjugation with ROS generation. Of note, the genetic knockdown of TSPO in human U118MG glioma cell line resulted in mitochondrial PPIX accumulation upon exposure of cells to PPIX, suggesting a role of TSPO in preventing intracellular accumulation of PPIX [71]. In addition, TSPO could act as a scavenger of porphyrin-based compounds in a eukaryotic model such as human colonic epithelial cell line (Caco-2) and may contribute to protecting cells from potential toxic compounds such as free tetrapyrroles [72]. Notably, TSPO ligands could antagonize the functions of the endogenous PPIX and for instance, PK11195 could counteract the cytotoxic effects of hemin in Caco-2 cells [72]. Employing detergent-purified bacterial *Chlorobium tepidum* TSPO, it was demonstrated that TSPO could induce rapid spectral changes to added PPIX indicative of chemical catalysis [73]. Moreover, in *Bacillus cereus*, TSPO mediated a light-induced degradation of PPIX [74]. Further, TSPO ligands were able to partially rescue cells from porphyrin-induced phototoxicity [75]. In *Arabidopsis thaliana*, it was observed that TSPO (AtTSPO) attenuated ALA-induced porphyria through a potential scavenging mechanism [64]. AtTSPO could possibly be involved in the transient clearance of excess cytosolic unbound heme and thereby it could modulate redox homeostasis [64]. However, the analysis of PPIX elimination in *Tspo*^−/−^ mouse tissues and plasma suggests that TSPO is not a critical regulator of PPIX levels in mammalian systems in normal physiological conditions [49]. Further, PPIX-mediated phototoxic cell death was not different between *Tspo^fl/fl^* and *Tspo*^−/−^ fibroblasts [49]. In addition, early studies using TSPO-binding pharmacological agents have suggested a functional link between mammalian TSPO and the induction of hemoglobin synthesis [76,77]. However, recent studies using *Tspo*^−/−^ mice and cell lines have established that TSPO is not involved in heme biosynthesis [49]. Though these recent observations rule out the role of TSPO in PPIX biosynthesis, given the evolutionarily conserved interaction between TSPO and porphyrins further studies are warranted elucidating the role of TSPO in oxidative stress associated with porphyrins including heme.

## 6. TSPO and Oxidative Stress

TSPO appears to be an essential participant in the regulation of mitochondrial reactive oxygen species (ROS) levels [78,79,80] and the mitochondrial location of the TSPO is interesting as mitochondria are the main source of cellular ROS [81]. Also, the exposure of neuronal cells to TSPO ligands in vitro generates oxygen-free radicals [82]. In the liver, TSPO was found in colocalization with the mitochondrial manganese-dependent superoxide dismutase, a ROS scavenger [83]. It has also been demonstrated that increased TSPO expression is associated with resistance against ROS and hydrogen peroxide cytotoxicity [84]. Along these lines, Jurkat cells transfected with *Tspo* cDNA exhibited higher resistance to free radical-mediated damage than controls [84]. Conversely, knockdown of TSPO augmented ROS production [25], suggesting that TSPO may participate in an antioxidant response pathway. Also, it has been suggested that TSPO could act to neutralize ROS. Along these lines, the tryptophan residues in TSPO might react with ROS to generate tryptophan radicals [74]. In MA-10 Leydig cells, CRISPR/Cas9-mediated deletion of TSPO resulted in a modest increase in ROS production compared to controls [52]. Further, it has been demonstrated that oxidative stress modulates both the structure and function of TSPO. Along these lines, increased ROS levels resulted in TSPO polymerization and enhanced ligand binding [85]. However, the precise role of TSPO in the regulation of cellular ROS levels or vice versa requires further studies.

## 7. TSPO and Neuroinflammation

**Figure 1:**
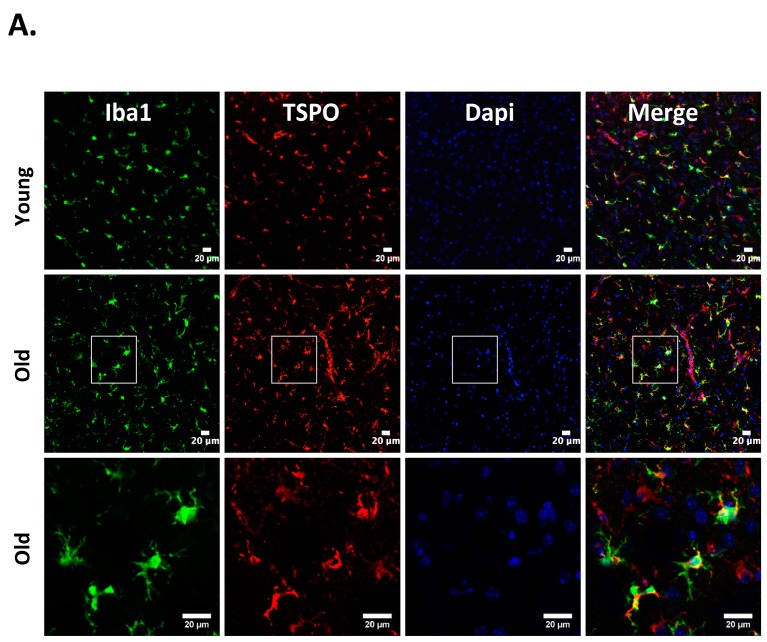

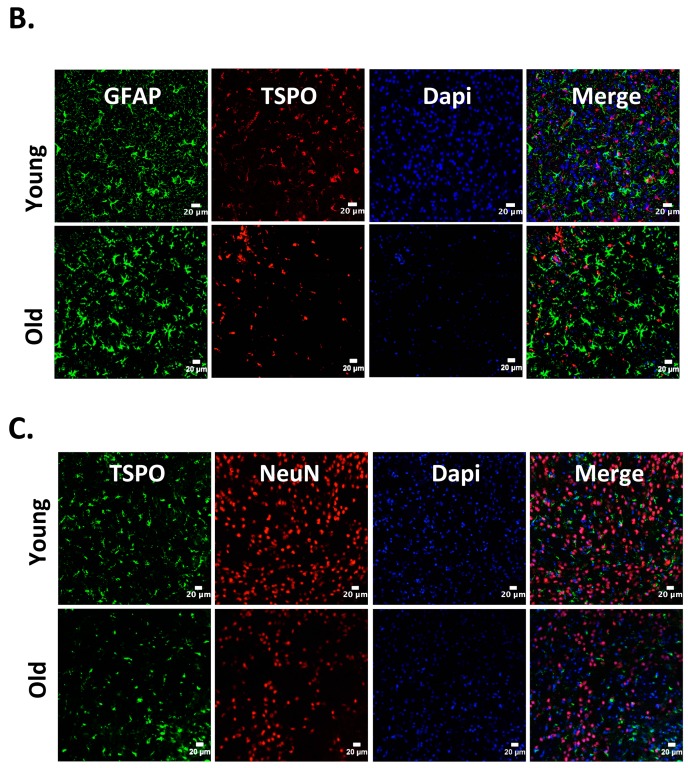
Cellular localization of 18kDa translocator protein (TSPO) expression in old vs. young mice after intracerebral hemorrhage (ICH). ICH was induced in male mice (20 months or 8 weeks old), as reported previously [27] and the brain sections were subjected to immunohistochemistry [27]. Briefly, brain sections were immunolabeled for (**A**) TSPO and Iba1 (microglia/macrophage marker); (**B**) TSPO and GFAP, (astrocyte marker) and (**C**) TSPO and NeuN (neuronal marker), 3 days-post ICH induction. A remarkable co-localization was observed between TSPO and Iba1, whereas no TSPO expression was observed in either NeuN or GFAP positive cells in both young as well as old mice. Scale Bar = 20 μM; *n* = 3 mice per group.

Neuroinflammation characterized by the activation of neuroimmune cells has been implicated as a pathological contributor to several neurodegenerative diseases. Under normal conditions, TSPO expression is low in immune-competent cells, macrophages, and leukocytes in the periphery, as well as in microglia and astrocytes [86]. In response to brain injury, the glial cells become activated and activated microglia/macrophage are often associated with increased expression of TSPO [18,19,87]. Therefore, TSPO is considered as a relevant molecular marker of neuroinflammation and could be an attractive therapeutic target. Though neuroinflammation is closely related to brain injuries and various neurodegenerative disorders such as Huntington’s disease, Dementia, Parkinson’s disease, and Multiple sclerosis, the precise functional consequences of microglial activation in these diseases are unclear. It is proposed that TSPO may regulate the release of pro-inflammatory cytokines during inflammation [27,88,89]. Consistently, genetic knockdown of TSPO in RAW 264.7 cells augmented hemin-induced release of proinflammatory cytokines revealing a negative regulatory role of TSPO in inflammation [27]. Further, our recent preclinical studies demonstrated an augmented expression of TSPO after intracerebral hemorrhage (ICH), a neuropathological condition that mostly afflicts the elderly population [27]. Importantly, irrespective of age, ≈85% TSPO expressing cells in the brain after ICH, co expressed Iba1 (microglia/macrophage marker) (Figure 1), implicating a possible role of TSPO in neuroinflammatory responses. Consistently, the expression of immune response genes was affected in TSPO1^−/−^ tissues [42]. Further, TSPO expression was also observed in activated microglia/macrophages of phagocytic phenotypes [27] and TSPO ligands induced the phagocytic capacity of microglia [90] suggesting an unexplored role of TSPO in microglia/macrophage-mediated phagocytosis in pathological conditions. Altogether, these studies suggest that TSPO may modulate microglia/macrophage, the inflammatory cells of the CNS, at multiple functional levels. Although both astrocytic, as well as microglial expression of TSPO, has been observed in various neuropathological conditions in rodents [26,91,92], microglia/macrophages but not astrocytes are the significant contributors of TSPO binding sites in human neuropathologies [93,94]. Of note, radiolabeled-TSPO ligands have been widely used for monitoring the augmented brain expression of TSPO in neuropathological conditions since TSPO-dependent enhanced binding of the radiotracer can be detected and quantified using non–invasive neuroimaging techniques such as positron emission tomography (PET) or Single-photon emission computed tomography (SPECT). Given the enhanced expression of TSPO in brain inflammatory cells, the neuroimaging employing the radioligands of TSPO provides a valuable tool allowing us to track and quantify the brain inflammation, and thereby ascertain the effectiveness of therapeutic interventions in a real-time manner. Along these lines, [11] C-labeled PK11195 is the first radiotracer that was used for the evaluation of activated microglia/macrophages and neuroinflammation in vivo. However, owing to the high lipophilicity, radiolabeled PK11195 exhibited high non-specific binding and a poor signal-to-noise ratio, complicating its quantification [95,96]. This has prompted the search for radiotracers with improved capacities to quantify TSPO expression. Along these lines, several new TSPO radioligands have been developed [97], and most of them have lower lipophilicity than radiolabelled-PK11195 and improved specific-to-nonspecific binding. Though microglia/macrophages are the most prominent cell type expressing TSPO in diseased brains, the mechanisms regulating augmented TSPO expression in microglia/macrophage, as well as the precise role of TSPO in microglia/macrophage functions in neuropathological conditions, remains largely unknown.

## 8. TSPO as a Therapeutic Target

Accumulating evidence implicate that synthetic TSPO ligands are neuroprotective in various CNS disorders [98] and thereby, TSPO is regarded as a therapeutic target for neurologic disorders [23,99]. Along these lines, Etifoxine, a TSPO ligand and clinically approved drug for the treatment of anxiety disorders, promoted axonal regeneration and functional recovery in an animal model of peripheral nerve freeze injury [100]. Further, both first and second-generation TSPO ligands, PK11195 and DPA-713 respectively, conferred neuroprotection against quinolinic acid injection into the rat striatum [101]. Interestingly, TSPO-agonist, Ro5-4864 significantly reversed the pathology associated with Alzheimer’s disease in vivo [102]. Though, one of the key mechanisms underlying the neuroprotective effects mediated by TSPO ligands has been implicated as the stimulation of mitochondrial steroid synthesis, the precise cellular and molecular mechanisms underlying TSPO ligand-mediated neuroprotection in neuropathological conditions are not well defined. However, in contrast to the neuroprotective role of ligand-mediated TSPO signaling as outlined above, the hGFAP-driven-conditional TSPO knockout mice exhibited reduced astrogliosis and experimental autoimmune encephalomyelitis clinical scoring in a preclinical mouse model of multiple sclerosis (MS) [103] and this discrepancy between the genetic and pharmacological studies demands a thorough investigation. Notably, the widely used TSPO agonist, Etifoxine binds and modulates GABAA receptors further implicating the need to establish the therapeutic potential of TSPO. Altogether, it is imperative to reassess the neuroprotective efficacy of TSPO ligands employing transgenic animal models, as it would validate the functional role of TSPO as a therapeutic target in various neuropathological conditions.

## 9. Conclusions

TSPO is an evolutionarily conserved protein with enigmatic functions. Apart from the identification of TSPO as a biomarker of glial activation future studies are warranted characterizing the precise role of TSPO in mitochondrial functioning as well as in cellular inflammatory and oxidative responses and it would also validate the therapeutic potential of TSPO in various pathological conditions.

## 10. Ethics Statement

Animal studies were reviewed and approved by the Committee on Biosafety and Animal Care and Use at Augusta University (protocol #2012-0459; 5 May 2015), in compliance with NIH and USDA guidelines.

## Abbreviations

TSPO	18 kDa translocator protein
GABA	gamma-aminobutyric acid
HUGO	human genome organization
CNS	central nervous system
ROS	reactive oxygen species
ALA	5-amino-laevulinic acid
CRISPR/Cas9	clustered regularly interspaced short palindromic repeats and CRISPR-associated protein 9
Iba1	ionized calcium binding adaptor molecule 1
GFAP	glial fibrillary acidic protein
DPA-713	*N*,*N*-diethyl-2-[2-(4-[methoxyphenyl)-5,7-dimethyl-pyrazolo-[1,5-α]pyrimidin-3-yl]-acetamide
GABAA receptors	gamma-aminobutyric acid type A receptors
